# Myocyte Enhancer Factor 2C as a New Player in Human Breast Cancer Brain Metastases

**DOI:** 10.3390/cells10020378

**Published:** 2021-02-12

**Authors:** Sofia Galego, Linda Azevedo Kauppila, Rui Malhó, José Pimentel, Maria Alexandra Brito

**Affiliations:** 1 Research Institute for Medicines (iMed.ULisboa), Faculty of Pharmacy, Universidade de Lisboa, Av. Prof. Gama Pinto, 1649-003 Lisbon, Portugal; sofiagalego11@hotmail.com; 2 Neurology, Department of Neurosciences and Mental Health, Hospital de Santa Maria, Centro Hospitalar e Universitário Lisboa Norte, Av. Prof. Egas Moniz, 1649-028 Lisbon, Portugal; linda.m.kauppila@gmail.com; 3 BioISI, BioSystems and Integrative Sciences Institute, Faculty of Sciences, Universidade de Lisboa, Campo Grande 016, 1749-016 Lisbon, Portugal; rmmalho@fc.ul.pt; 4 Faculty of Medicine, Universidade de Lisboa, Av. Prof. Egas Moniz, 1649-028 Lisbon, Portugal; josepimentel@medicina.ulisboa.pt; 5 Laboratory of Neuropathology, Department of Neurosciences and Mental Health, Hospital de Santa Maria, Centro Hospitalar e Universitário Lisboa Norte, Av. Prof. Egas Moniz, 1649-028 Lisbon, Portugal; 6 Departamento de Ciências Farmacêuticas e do Medicamento, Faculty of Pharmacy, Universidade de Lisboa, Av. Prof. Gama Pinto, 1649-003 Lisbon, Portugal

**Keywords:** breast cancer brain metastases, biomarker, β-catenin, myocyte enhancer factor 2C, proliferation, vascular endothelial growth factor receptor-2

## Abstract

Myocyte enhancer factor 2C (MEF2C) is increasingly expressed in mice along with breast cancer brain metastases (BCBM) development. We aim to ascertain MEF2C expression in human BCBM, establish the relationship with disease severity, disclose the involvement of vascular endothelial growth factor receptor-2 (VEGFR-2) and β-catenin, also known as KDR and CTNNB1, respectively, and investigate if matched primary tumors express the protein. We studied resected BCBM for the expression of MEF2C, VEGFR-2, and ß-catenin, as well as proliferation (Ki-67) and epithelial (pan Cytokeratin) markers, and related experimental and clinical data. MEF2C expression was further assessed in matched primary tumors and non-BCBM samples used as controls. MEF2C expression was observed in BCBM, but not in controls, and was categorized into three phenotypes (P): P1, with extranuclear location; P2, with extranuclear and nuclear staining, and P3, with nuclear location. Nuclear translocation increased with metastases extension and Ki-67-positive cells number. P1 was associated with higher VEFGR-2 plasma membrane immunoreactivity, whereas P2 and P3 were accompanied by protein dislocation. P1 was accompanied by β-catenin membrane expression, while P2 and P3 exhibited β-catenin nuclear translocation. Primary BC samples expressed MEF2C in mammary ducts and scattered cells in the parenchyma. MEF2C emerges as a player in BCBM associated with disease severity and VEGFR-2 and β-catenin signaling.

## 1. Introduction

Breast cancer (BC) is the most frequent malignant tumor and the leading cause of cancer death among women, with 2.1 million new cases and 626,679 deaths in 2018 [1]. With improvements in primary tumor treatments, patient survival has increased, witnessing the development of metastases in 15–30% of BC patients [2,3]. Brain metastases (BM) limit both life expectancy and quality of life (QoL), being the main cause of death in BC patients, with an annual incidence of 8.3–14.3 per 100,000 individuals [4,5]. However, the mechanisms associated with BC brain metastases (BCBM) development are still unclear, rendering the unraveling of specific tumor molecular pathways necessary to develop innovative targeted therapeutics.

BC usually starts in mammary duct epithelial cells that lose their epithelial properties and gain mesenchymal characteristics, leading to the loss of adhesion and acquisition of invasive properties. This process, known as an epithelial–mesenchymal transition (EMT), allows the invasion of surrounding tissues and metastases development [6]. BC cells (BCCs) constitute a heterogeneous population that can be classified based on the expression of human epidermal growth factor receptor 2 (HER2), progesterone receptor (PR), and estrogen receptor (ER), as ER+/PR+, HER2+; ER+/PR+, HER2−; ER−/PR−, HER2+; and triple negative (TN), which is characterized by the absence of ER, PR, and HER2 [7,8], and presents the worst prognosis and lowest overall survival [9,10]. BCCs migrate differently to secondary organs depending on the BC molecular subtype [11], with HER2 and TN subtypes being highly associated with brain metastases formation [12,13,14].

Myocyte enhancer factor 2 (MEF2) family of transcription factors (TFs), composed of MEF2A, MEF2B, MEF2C, and MEF2D, play crucial roles in organ development and tissue differentiation [15] and are expressed in muscle, neuronal, chondroid, immune, and endothelial cells [16]. MEF2 TFs regulate epigenetic modifications and control gene expression by activation or repression of transcription depending on the establishment of interactions with co-activators (e.g., p300) or co-repressors (e.g., class IIa histone deacetylases). Thus, MEF2 family members regulate signaling pathways during normal and pathological conditions, including cancer. However, due to the duality of effects, the role of MEF2 TFs in cancer is still controversial, rendering difficult their unequivocal categorization as oncogenes or tumor suppressors. Moreover, the distinct but also overlapping functions and expression patterns among the four family members in different tissues, lineages, and differentiation stages renders the transcriptome under MEF2 regulation heterogeneous [15]. In fact, studies by Clocciatti et al. [17] reported that MEF2A and MEF2D are the most expressed in breast tissue and present a growth repressive effect, decreasing mitotic activity. In contrast, Ostrander et al. [18] reported that MEF2C is expressed in normal mammary epithelial cells and breast cancer cell lines, and Schuetz et al. [19] further identified the *MEF2C* gene in ductal carcinoma in situ and invasive ductal carcinoma. However, variable effects of MEF2C expression levels on BC patient survival as a function of disease time, race, and type of breast cancer, with the worst prognosis corresponding to Luminal BC with high MEF2C, are presented in the UALCAN database (http://ualcan.path.uab.edu; accessed in 24 January 2021). The regulation of MEF2 transcription is determined by several signaling pathways, including MAP kinases, Wnt/β-catenin, PI3K/Akt, and Ca^2+^ signaling, with microRNAs also contributing to regulate MEF2 activities [20]. Moreover, a number of MEF2 targets have been identified, namely related to cancer (e.g., *MYC*, *TGFB1*, *CARD11*, *RHOB,* and *NDRG1*) [21], and particularly with sprouting angiogenesis in tumor vascularization (e.g., *Delta-like ligand 4*, *Dll4*) [22].

Previous studies from our laboratory using a mouse model of brain metastases development from triple negative breast cancer (TNBC) revealed that microRNAs 802-5p and 194-5p are downregulated in plasma prior to detection of BM from BC and that MEF2C is a target of both miRNAs. Such studies further revealed that MEF2C is expressed in BCCs extravasating into the brain and in well-established metastases and that it is increasingly translocated into the nucleus as tumorigenesis progresses [23]. Due to MEF2C function as a TF, it is conceivable that this translocation culminates in higher activation of MEF2C function to promote the transcription of its target genes, such as *vascular endothelial growth factor (VEGF)* [18,24]. VEGF is an inducer of angiogenesis, which is crucial for tumor growth, invasion, and metastasis development [25]. VEGF binds to the VEGF receptor 2 (VEGFR-2), also known as kinase domain insert domain receptor (KDR), activating MEF2C [26]. VEGF signaling is induced by nuclear MEF2C, increasing angiogenesis and tumor invasion [24]. On the other hand, cytosolic MEF2C has been shown to inhibit tumor growth via Wnt/β-catenin (CTNNB1) signaling, which is involved in the regulation of cancer cell proliferation [24]. This indicates that MEF2C presents a dual role in tumor development depending on its cellular location and points to MEF2C as a new player in BCBM that deserves to be better unraveled, together with its partners VEGFR-2 and β-catenin.

In this study, we aimed to establish whether MEF2C is expressed in human BCBM, and its association with relevant cancer-associated signaling pathways, such as VEGF and β-catenin, and whether its expression is tumor-specific. We analyzed resected BM samples from BC patients for the expression of MEF2C, VEGFR-2, and ß-catenin, as well as for proliferation (Ki-67) and epithelial/endothelial (pan Cytokeratin) markers, which allowed us to establish a relationship between MEF2C expression pattern and disease severity. Further analysis of MEF2C and pan Cytokeratin in brain tissue used as control revealed that MEF2C is nearly absent in non-BCBM tissue, pointing to its disease-specific expression. Moreover, an inspection of MEF2C and the epithelial (pan Cytokeratin) or mesenchymal (vimentin) markers in matched primary tumors revealed its exuberant expression in mammary ducts cells and some scattered cells in the parenchyma, pointing to MEF2C as a tumorigenesis indicator.

## 2. Materials and Methods

### 2.1. Patients

Archived formalin-fixed paraffin-embedded (FFPE) resected BCBM and primary BC tissues available at the Laboratory of Neuropathology and at the Department of Pathology of Hospital de Santa Maria, Lisbon, Portugal, respectively, were used. Tissue was obtained in a manner compliant with the Declaration of Helsinki, as revised in 1983. Patients’ data were extracted retrospectively from electronical clinical records and reviewed by members of the responsible clinical team, in compliance with data protection and in accordance with local ethics committee regulations.

The study involved neoplastic tissue from 24 cases of BCBM and 3 paired resected primary BC. Samples were derived from women aged 29–78 years old, with simple or multiple BCBM, whose size ranged between 1 and 6 cm. Most cases corresponded to primary tumors HER2+ and/or ER+/PR+ (Table 1). Moreover, 10 resected brain tissues derived from patients with diffuse gliomas (Table 2), distant from the invasion/lesioned areas, were analyzed as non-BCBM controls. Hematoxylin-eosin staining revealed no reactive or neoplastic astrocytes and microglia, providing as much certainty as possible of the tissue integrity.

### 2.2. Immunofluorescence

Three µm-thick sections were processed for immunofluorescence (IF) analysis of MEF2C, pan Cytokeratin, Ki-67, VEGFR-2, and β-catenin expression in BCBM, as well as of MEF2C and pan Cytokeratin in controls, and of MEF2C with pan Cytokeratin or vimentin in BC primary tumors. Sections were deparaffinized, rehydrated, subjected to heat mediated antigen retrieval, and permeabilized. Afterward, sections were blocked and incubated with the primary and secondary antibodies (Table 3). Negative controls with the omission of the primary antibody were performed to exclude nonspecific binding or cross-reactivity, and nuclei were labeled with Hoechst 33342 dye (ThermoFisher Scientific, Waltham, MA, USA; 1:1000).

### 2.3. Data Analysis

Images were acquired at the Faculty of Sciences, University of Lisbon Microscopy Facility, a node of the Portuguese Platform for BioImaging (PPBI-POCI-01-0145-FEDER-022122), using a fluorescence microscope (Olympus, Tokyo, Japan, BX60) with a mercury fluorescence illuminator, and a Nomarski/DIC Prism for Transmitted Light, or at the Faculty of Pharmacy, University of Lisbon, using an AxioScope.A1 microscope (Zeiss, Oberkochen, Germany), equipped with a Leica DFC 490 camera.

Ten fields per section were acquired using the 40× objective and were analyzed using ImageJ 1.29x software (National Institutes of Health, Bethesda, MD, USA).

BCBM were analyzed for MEF2C expression in metastatic cells based on double labeling with pan Cytokeratin, as well as for its nuclear translocation [23]. The MEF2C expression pattern was related to clinical data, and results were presented as a percentage of cases with a certain MEF2C expression pattern (cytosol/nucleus) as a function of the metastases size (1–≤3 and 3–6 cm diameter) and number (solitary or multiple). To analyze the cancer cell proliferation, the number of Ki-67-positive cells per metastasis was counted. To evaluate the involved signaling pathways, the number of cells with nuclear ß-catenin staining was expressed as a function of the total number of cells in each metastasis. The VEGFR-2 immunoreactivity was analyzed by measuring VEGFR-2 mean intensity per metastatic cell. Expression of MEF2C was also assessed in BC primary tumors based on double labeling with pan Cytokeratin or vimentin.

### 2.4. Statistical Analysis

The results are expressed as mean ± SD. Results were analyzed using GraphPad Prism^®^ 5.0 (GraphPad Software, San Diego, CA, USA). One-way ANOVA and the Bonferroni post hoc test were used to evaluate statistically significant alterations between different phenotypes, and the chi-square test was performed to assess the association between the phenotypes and the number and size of metastases. *p*-values less than 0.05 were considered statistically significant.

## 3. Results

### 3.1. MEF2C Expression Pattern in BCBM

**Figure 1:**
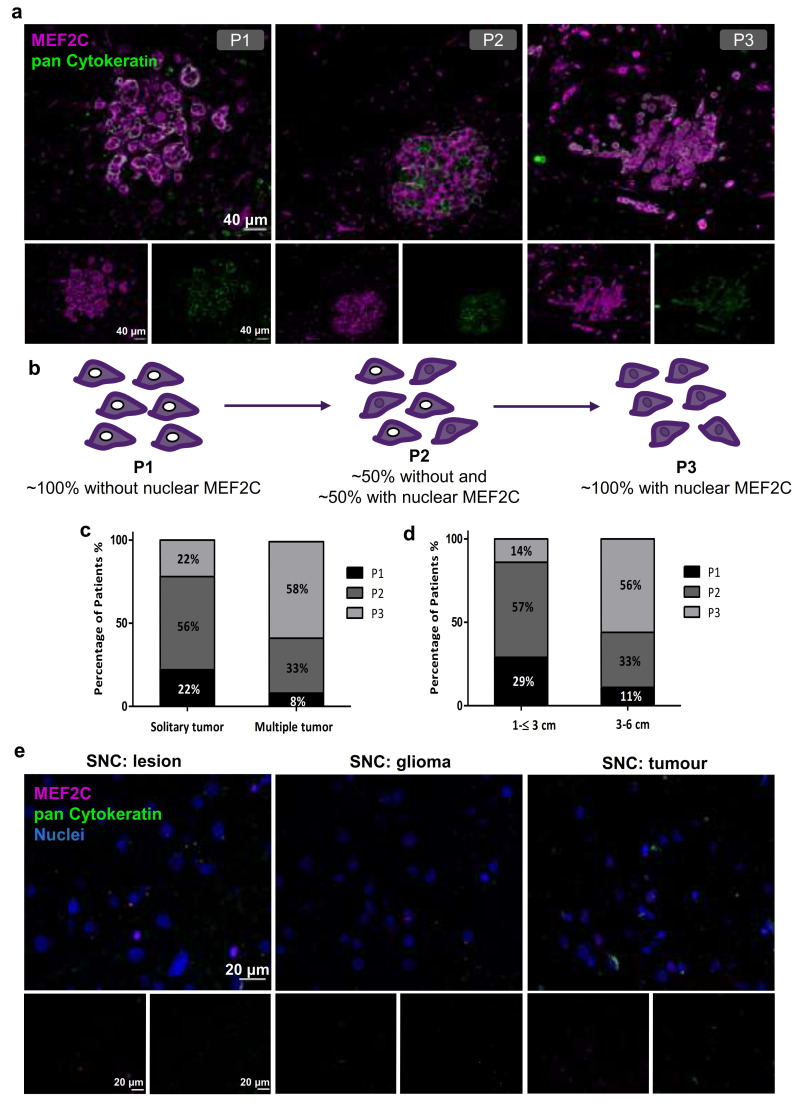
Myocyte Enhancer Factor 2C expression in resected human brain metastases derived from breast cancer patients and from glioma patients. Immunofluorescence analysis of myocyte enhancer factor 2C (MEF2C) (purple) and of the epithelial and tumoral marker, pan Cytokeratin (green), in human brain metastases from breast cancer patients, revealed distinct MEF2C labeling patterns that were considered as three different phenotypes: ~100% cells presenting an extranuclear location (P1); ~50% cells presented extranuclear location and ~50% presented overall cell staining (P2); or ~100% cells presenting overall cell staining (P3) (**a**). Schematic representation of the subcellular MEF2C distribution, according to the considered phenotypes (P1, P2, P3) (**b**). Semi-quantitative analysis of the percentage of patients presenting each phenotype, regarding the number (**c**) and the size of the metastases (**d**), shows the progression from P1 to P3 as the number and size of metastases increase. Double labeling of MEF2C and pan Cytokeratin in brain tissue samples derived from glioma patients as non-breast cancer brain metastases control (**e**) showing no relevant expression of the protein. Twenty-four cases of BCBM (P1, n = 3; P2, n = 10; P3, n = 11) and ten control cases were studied; ten fields per section and one section per case were analyzed.

To understand if MEF2C expression and nuclear translocation at later stages of tumorigenesis observed in our previous mouse studies [23] are translatable to humans, we analyzed MEF2C expression in resected BM from women with BC. To assess whether cells expressing MEF2C are tumor cells, double staining was performed with an epithelial tumor marker, pan Cytokeratin [6]. We found that BCBM expressed MEF2C (Figure 1). Interestingly, several MEF2C labeling patterns, corresponding to different cellular locations, were detected. This led to the categorization of patients into three phenotypes (P): P1, with MEF2C extranuclear location; P2, where ~50% cells presented extranuclear location and ~50% presented overall cell staining; and P3, where 100% cells presented overall cell staining (Figure 1a,b). Within the 24 studied cases, 3 (12.5%) exhibited P1, 10 (41.7%) displayed P2, and 11 (45.8%) presented P3. Further, we analyzed if these phenotypes were associated with the metastases number (solitary or multiple) and tumor size (1–≤ 3 or 3–6 cm). We observed that the increase in metastases number was accompanied by a decrease in the percentage of cases with P1 (from 22 to 8%) and P2 (from 56 to 33%), and an increase in the percentage of P3 cases (from 22 to 58%) (Figure 1c). Similarly, a decrease in the percentage of cases with P1 (from 29 to 11%) and P2 (from 57 to 33%) together with an increase in cases with P3 (from 14 to 56%) occurred as the tumor size increased (Figure 1d). These results point to an association between the nuclear translocation of MEF2C and the severity of tumorigenesis. Accordingly, a trend to statistical significance was observed for metastases number and size as MEF2C assumes a nuclear location (*p* = 0.0980 and *p* = 0.0907, respectively, chi-square test, performed for P1 + P2 versus P3).

To understand whether the MEF2C expression pattern was specific to BCBM, we analyzed 10 brain tissue samples derived from glioma patients as non-BCBM controls. IF staining revealed that MEF2C expression is nearly absent in these non-BCBM controls (Figure 1e), suggesting that the TF expression observed in human BCBM is specific to this disease.

### 3.2. MEF2C Expression in BC Primary Tumours

**Figure 2:**
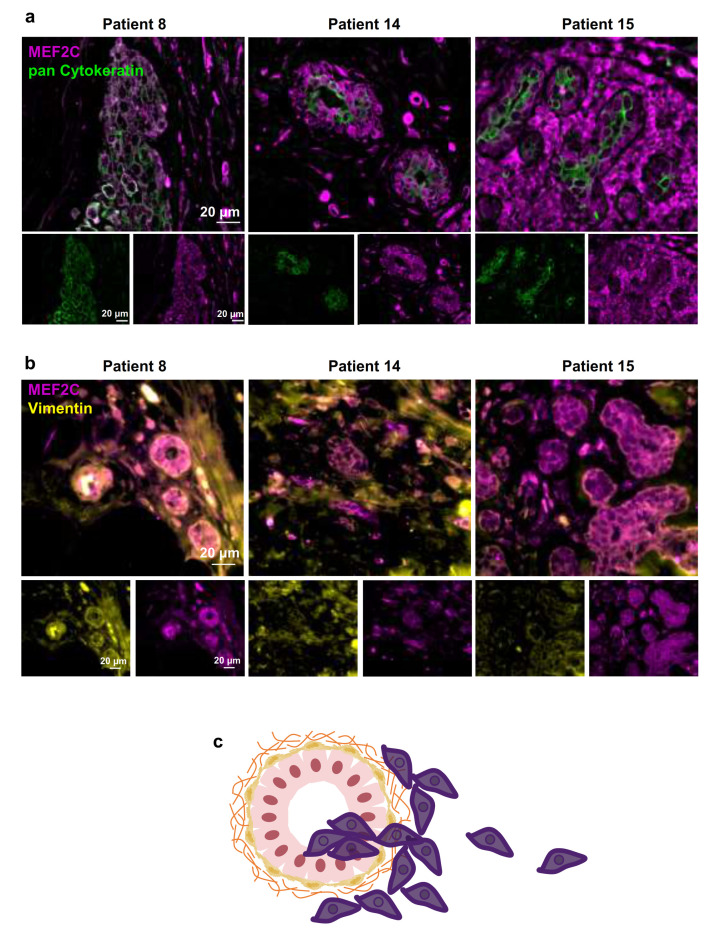
Myocyte Enhancer Factor 2C expression in human breast cancer primary tumors. Double immunofluorescence analysis of myocyte enhancer factor 2C (MEF2C; purple) with the epithelial and tumoral marker, pan Cytokeratin (green), showed that MEF2C expressing cells did not significantly express pan Cytokeratin and were found in disorganized mammary ducts as well as in the surrounding tissue (**a**). Double immunofluorescence analysis of MEF2C (purple) with the mesenchymal marker, vimentin (yellow), showed that MEF2C expressing cells in mammary ducts also expressed vimentin (**b**). Schematic representation of the first stages of the metastatic cascade, showing MEF2C expressing cells (purple) in the mammary duct and invading the surrounding tissue (**c**). Three resected human primary BC cases were studied; ten fields per section and one section per case were analyzed.

The metastatic cascade is composed of sequential events that allow the dissemination of cancer cells from the primary tumor to other secondary organs, such as the brain [27]. To investigate if malignant cells express MEF2C at early steps of the metastatic cascade, we double-labeled MEF2C with pan Cytokeratin or with vimentin in paired BC primary tumors corresponding to BCBM with MEF2C P3 (Figure 2). We observed MEF2C expression in disorganized ductal cells, and in scattered cells located outside mammary ducts with poor pan Cytokeratin expression (Figure 2a), as well as in ductal cells expressing vimentin (Figure 2b). Our findings indicate that MEF2C is expressed by primary BC tumor cells either located in ducts or the parenchyma, as represented in Figure 2c. To confirm that MEF2C is expressed by BCCs, analysis of the protein and the corresponding mRNA was performed in 4T1 cells, a mouse TNBC cell line. As shown in Supplementary Figure S1, MEF2C is consistently expressed in the malignant cells, and the mRNA levels decreased up to 80% by silencing with MEF2C siRNA. Further validation was performed by analysis of the protein in MDA-MB-231 Br4, a human cell line with brain tropism (Supplementary Figure S2), which revealed a clear MEF2C expression in these BCCs with proneness to form brain metastases. Moreover, it revealed that the protein is present not only in the cytosol but also in the nuclei, corroborating the nuclear translocation described above. The observation that the cultured cells express the epithelial marker pan Cytokeratin and, more markedly, the mesenchymal one, vimentin, is also interesting. These observations are in line with those of tissue sections showing that several MEF2C-positive cells did not exhibit a strong expression of pan Cytokeratin but revealed a sustained vimentin presence (Figure 2a,b).

### 3.3. Influence of MEF2C Cellular Location in Cancer-Associated Signaling Pathways

**Figure 3:**
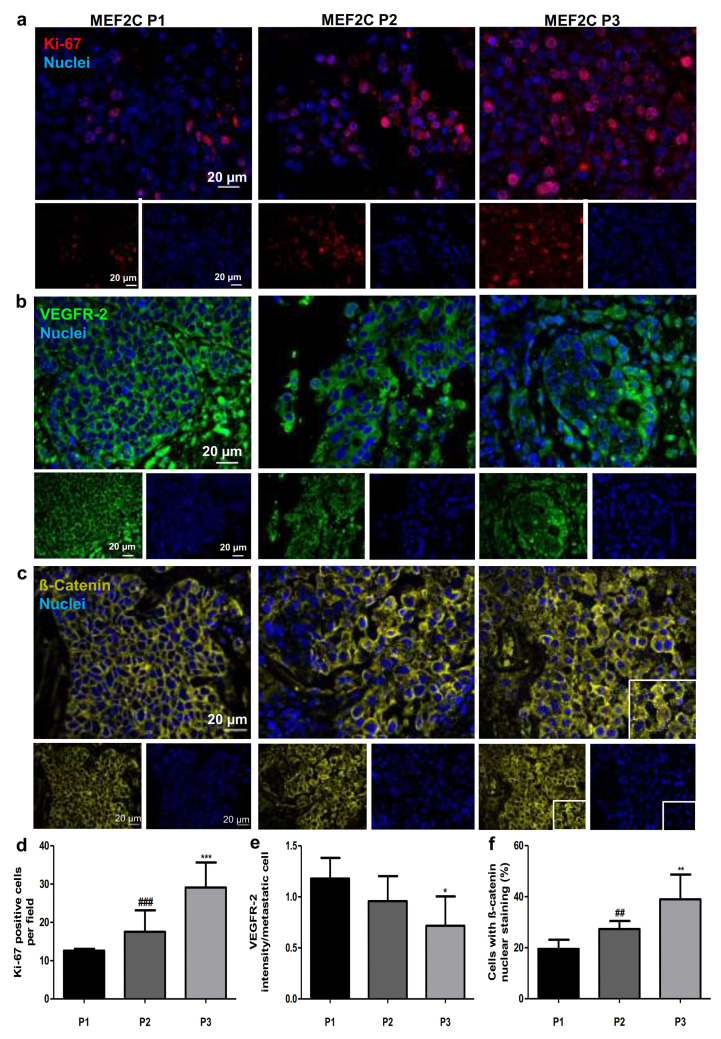
Ki-67, vascular endothelial growth factor receptor 2 (kinase domain insert domain receptor, KDR) and ß-catenin (CTNNB1) expression in resected human breast cancer brain metastases. Immunofluorescence analysis was performed in tissue sections from breast cancer brain metastases. Analysis of the proliferation marker, Ki-67 (red), revealed an increasing presence of Ki-67 positive cells in breast cancer brain metastases as a function of the myocyte enhancer factor 2C (MEF2C) expression phenotype (P1, P2 and P3) (**a**). Analysis of vascular endothelial growth factor receptor 2 (VEGFR-2; green) revealed a loss of membrane expression and a decreased intensity of this receptor with an increase in MEF2C nuclear translocation (**b**). Analysis of the downstream effector of Wnt cascade, ß-catenin (yellow), revealed an increasing number of cells with ß-catenin nuclear staining as MEF2C expression phenotype progressed from P1 to P3 (**c**). Semi-quantitative analysis of Ki-67 positive cells (**d**), VEGFR-2 total intensity (**e**), and percentage of cells with nuclear ß-catenin/metastasis (**f**) showed progressive variations accordingly with MEF2C phenotypes. Twenty-four cases of BCBM were studied; ten fields per section and one section per case were analyzed. Data are mean ± SD. One-way ANOVA and the Bonferroni post hoc test were used to evaluate statistically significant alterations between different phenotypes. Statistical analyses are denoted as * *p* < 0.05, ** *p* < 0.01, *** *p* < 0.001 vs. P1 and ^##^ *p* < 0.01 and ^###^ *p* < 0.001 for differences between P2 and P3.

MEF2C was associated with VEGF and Wnt/β-catenin signaling pathways in hepatocellular carcinoma [24]. To study if these cascades are involved in BCBM, we analyzed Ki-67, a proliferation marker, as well as VEGFR-2, a key player of VEGF pathway, and ß-catenin, a downstream effector of Wnt pathway, in resected BCBM (Figure 3). We observed a clear proliferation activity in all cases and a differential Ki-67 expression depending on the MEF2C subcellular location (Figure 3a). In fact, the number of Ki-67 positive cells showed a ~2.3-fold increase between P1 and P3 (*p* < 0.001) and a ~1.7-fold elevation between P2 and P3 (*p* < 0.001) (Figure 3d).

Analysis of VEGFR-2 labeling showed a clear expression of the receptor at the cell membrane in MEF2C P1 samples, which shifted into the cytoplasm in P2 and P3 (Figure 3b). Semi-quantitative analysis of the immunoreactivity in metastatic cells showed that the highest intensity of VEGFR-2 in metastases occurred when MEF2C location was extranuclear (P1) (Figure 3e) and corresponded to the expression of the receptor at the membrane level (Figure 3b). Note that the shift from the membrane to cytoplasm immunostaining observed from P1 to P2 was not accompanied by a statistically significant decrease in VEGFR-2 expression, which was only noticed in P3 as compared with P1 (0.4-fold decrease, *p* < 0.05) (Figure 3e).

Similar to VEGFR-2, a change in the ß-catenin expression pattern was observed, with a progressive shift of protein location in the membrane in P1, to the cytosol and the nucleus in P2 and P3, respectively (Figure 3c). In fact, the percentage of BCCs presenting nuclear ß-catenin increased ~2.0-fold from P1 to P3 (*p* < 0.01) and ~1.4-fold from P2 to P3 (*p* < 0.01) (Figure 3f).

**Figure 4:**
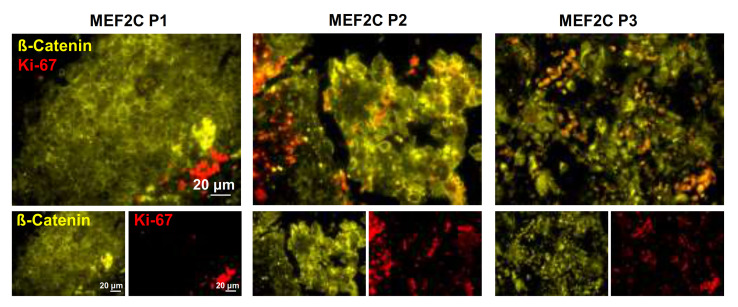
Ki-67 and ß-catenin (CTNNB1) expression in resected human breast cancer brain metastases. Double-labeling immunofluorescence analysis was performed in tissue sections from breast cancer brain metastases representative of each myocyte enhancer factor 2C (MEF2C) expression phenotype (P1, P2, and P3). Analysis of the proliferation marker Ki-67 (red) in metastases labeled with β-catenin (yellow) revealed the presence of Ki-67 positive cells at the periphery of metastasis in which β-catenin was mainly expressed at the cell membrane (corresponding to MEF2C P1), as well as an increasing number of Ki-67 positive cells and their presence inside metastases as β-catenin dislocated to the cytosol and nucleus (corresponding to MEF2C P2 and P3). Ten fields of one representative section of each MEF2C phenotype were analyzed.

To reinforce the relationship between MEF2C nuclear translocation in BCBM development and associated signaling molecules, double labeling of Ki-67 and β-catenin was performed. As shown in Figure 4, an increasing number of Ki-67-positive cells alongside β-catenin subcellular dislocation was observed. Moreover, the positive cells for Ki-67 were essentially observed at the periphery in metastases with β-catenin mostly expressed at the cell membrane (MEF2C P1), whereas in metastases exhibiting β-catenin shifting into the cytosol and nucleus (MEF2C P2 and P3), the total number of Ki-67 increased, together with the presence of such proliferating cells inside the metastases. Interestingly, cultures of TNBC cells with proneness to metastasize into the brain (MDA-MB 231 Br4) that expressed MEF2C (Supplementary Figure S2) were also positive for the proliferation marker Ki-67 and expressed both VEGFR-2 and β-catenin (Supplementary Figure S3). Thus, the observations in resected brain metastases together with those of cultured cells, reinforce the proposed association between MEF2C, VEGFR-2 and β-catenin signaling and the proliferative status of malignant cells.

## 4. Discussion

Compared to other secondary organs, the knowledge about the mechanisms responsible for BCBM formation and the development of treatment strategies remains limited [28,29]. Considering that BCBM represent a serious oncologic problem and are the leading cause of death of BC patients, the discovery of prognostic biomarkers of BC patients presenting BM and of novel therapeutic targets is imperative. This study showed that MEF2C is expressed in resected BCBM, with a variable pattern according to disease severity, which points to this TF as a potential prognostic biomarker. Moreover, it revealed the presence of MEF2C in matched primary tumours, particularly in mammary duct cells that lost their epithelial features and in scattered parenchymal cells, indicating that its detection in mammary biopsies and/or resected tumors may constitute an alert for the risk of metastases development. Finally, the alterations in VEGFR-2 and β-catenin as a function of MEF2C subcellular distribution points to a signaling axis in which these players are involved, and that can be targeted for the prevention of BCCs proliferation and abrogation of BM.

There are several evidences of MEF2C involvement in tumor progression [15]. In fact, MEF2C was proposed to promote metastases development in pancreatic adenocarcinoma by inducing metalloproteinase (MMP) 10 transcription [30] and to promote myeloid leukaemia, behaving as an oncogene by cooperating with the Sox4 gene [31]. Data showing its upregulation in colorectal cancer during disease progression and its association with BC invasion support a MEF2C pro-oncogenic function [19,32]. On the other hand, in hepatocellular carcinoma, MEF2C was overexpressed, mediating VEGF induction of vasculogenic mimicry, migration, and invasion, and presenting both oncogenic and tumor-suppressive properties [24,33]. Despite its recognized functions in several types of cancer development and progression, the role of MEF2C in BC, and particularly in BCBM, is still unexplored, raising interest in its study in BCBM. In this context, our previous studies in a mouse model showed that MEF2C is increasingly expressed along with BM development, and that it is expressed in scattered malignant cells located along brain capillaries, suggesting that malignant cells extravasating into the brain parenchyma already express the TF [23]. Moreover, the protein was progressively translocated into the nucleus as BM developed, compatible with TF activation.

Here, analysis of resected brain tissues from BC patients that developed BM revealed MEF2C overexpression in pan Cytokeratin-positive cells, reflecting the expression of this TF in malignant cells colonizing the brain. It further suggested different expression profiles exhibited by MEF2C-positive cells depending on protein subcellular location, which led us to categorize it into three different phenotypes. We observed that cases with higher metastases number and greater tumor size presented the P3 phenotype, corresponding to nuclear translocation of MEF2C in all cells, indicating an association between MEF2C nuclear translocation and disease severity. Moreover, MEF2C expression seems to be BCBM-specific as almost no expression of this TF was observed when resected brain samples from glioma were analyzed as non-BCBM controls by focusing in regions with no reactive or neoplastic astrocytes and microglia to assure tissue integrity as much as possible.

Since MEF2C nuclear translocation was reported to promote the transcription of its target genes *vegf* and *mmp10* [24,30], MEF2C seems to have an important role in cancer-associated signaling pathways. Here, we also observed the expression of players involved in VEGF and Wnt/ß-catenin signaling pathways, such as VEGFR-2 and ß-catenin, and further observed alterations in their expression pattern, according to different MEF2C phenotypes. We found that VEGFR-2 immunoreactivity in BM changes with the MEF2C phenotype. In fact, VEGFR-2 staining at the cell membrane observed in samples with MEF2C P1 (mostly in the cytosol) shifted to a cytoplasm staining in samples with MEF2C P2 and P3 (increasing nuclear translocation), suggesting that the membrane receptor is internalized as the disease severity increases. Such internalization would be in line with the previous suggestion of constitutive endocytosis (in the absence of the ligand, VEGF) that would protect the receptor against shedding, this way regulating the activity of the growth factor receptor [34]. Once in the cytoplasm, VEGF receptors may be either recycled back to the membrane or shuttled to lysosomes for degradation [35]. In advanced BCBM, increasing VEGFR-2 degradation appears to occur as the overall staining intensity/cell was lower in the group of patients presenting the most severe MEF2C phenotype (P3), which, in turn, was associated with the highest level of proliferative cells (Ki-67-positive). Interestingly, our recent studies in a mouse model of BCBM revealed that the microvascular density peaked at 7 days post-tumor cells injection, decreasing thereafter (Figueira et al., submitted). This observation suggests that the VEGF/VEGFR-2 signaling may somehow be altered in advanced stages of the brain metastatic process and is in line with the presently suggested VEGFR-2 degradation in P3 patients.

Previous studies reported MEF2Cs inhibitory role in ß-catenin nuclear translocation, leading to the inhibition of the Wnt/ß-catenin signaling pathway, which is responsible for cancer processes, such as metastases formation and tumor growth [24,36]. Our results revealed that ß-catenin is located at the cell periphery in BM with MEF2C located in the cytosol (P1), rather than in the nucleus. In contrast, an increase in nuclear ß-catenin was observed in patients with MEF2C P3, presenting the highest level of proliferative cells, pointing to an association of disease severity with the increase in nuclear ß-catenin and Ki-67 expression. These findings raise the hypothesis that in the early stages of metastases development, cytosolic MEF2C inhibits Wnt/ß-catenin signaling by impairing ß-catenin translocation into the nucleus, in contrast with advanced stages, where MEF2C is translocated into the nucleus and β-catenin also shifts to this subcellular compartment.

The metastatic process comprises sequential events that culminate with the dissemination of cancer cells from primary tumors to secondary organs [27]. Initially, BCCs undergo EMT, losing their epithelial and adhesion characteristics while acquiring mesenchymal features that endow them with invasive properties [6]. Schuetz et al. showed that MEF2C is one of the proteins related to the BC invasion process [19]. Interestingly, we found MEF2C expression in primary BC tumors, mainly in disorganized ductal cells and scattered cells located outside mammary ducts, which presented a poor or no expression of the epithelial and tumoral marker, pan Cytokeratin. These observations suggest that MEF2C-expressing cells may have lost the epithelial characteristics and are endowed with motility properties that are essential for metastases occurrence [6], which is in line with the expression of the mesenchymal marker vimentin. Interestingly, malignant cells continually expressed MEF2C in established brain metastases, which exhibited pan Cytokeratin expression. This indicates that metastatic cells colonizing the brain reacquire the original epithelial features, which is crucial for the development of brain neoplasms and is known as mesenchymal to epithelial transition (MET) [37]. Therefore, MEF2C expression occurs not only in the initial steps of the metastatic cascade but also in the brain colonization, with a sustained expression alongside the loss and regain of epithelial features.

As a retrospective study relying on human brain tissue analysis, the current study has some inherent limitations. The impossibility of obtaining healthy human brain tissue required the use of brain samples from diseases other than BCBM to establish that MEF2C is not widely expressed in the brain. The fact that BCBM frequently occur several years after the primary tumor is diagnosed and that the studied BCBM patients were treated for the primary tumor in different hospitals rendered the number of primary BC samples paired with the BCBM available small. Finally, the size of the studied BCBM cohort was modest.

## 5. Conclusions

**Figure 5:**
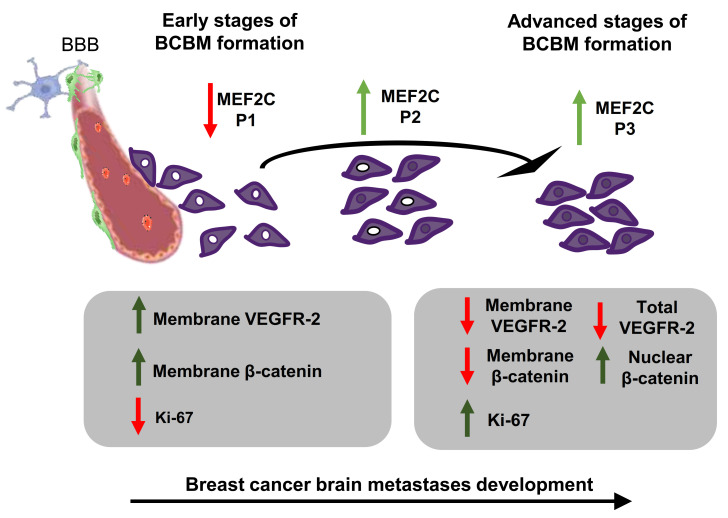
Schematic representation of the proposed interplay between myocyte enhancer factor 2C, vascular endothelial growth factor receptor 2 (kinase domain insert domain receptor, KDR), and β-catenin (CTNNB1) along with the development of breast cancer brain metastases. In the initial stages of brain metastases, the pattern of myocyte enhancer factor 2C (MEF2C) expression in malignant cells extravasating into the brain and still close to blood–brain barrier (BBB) microvessels is categorized as phenotype (P) 1 (~100% cells with extranuclear location). As metastases progress, the transcription factor progressively translocates into the nucleus giving rise to P2 (~50% cells with extranuclear location and ~50% with overall cell staining), and afterward, to P3 (~100% cells presenting overall cell staining). In parallel, the clear expression of vascular endothelial growth factor receptor 2 (VEGFR-2) at the cell membrane decreases, and an overall loss of the receptor is observed as metastases develop. This is accompanied by a decreased expression of β-catenin in the plasma membrane, which translocates into the nucleus, in line with the role of this molecule in signaling in cancer. Accordingly, an increasing number of Ki-67 positive cells is observed, reflecting the proliferative activity of metastatic cells that accounts for the enlargement of metastases. Collectively, the expression of MEF2C and its translocation into the nucleus is associated with disease severity, which pathogenesis involves VEGFR-2 and β-catenin signaling.

The findings reported in this study show that MEF2C was consistently expressed in BCBM and that its nuclear translocation was related to brain metastatic disease severity, based on clinical data and corroborated by analysis of proliferative cells. Moreover, they established associations between MEF2C expression patterns and VEGFR-2 and β-catenin status, adding to the current knowledge of the signaling molecules playing a role in BCBM development. Additionally, demonstration of MEF2C expression exhibited in the mammary tissue of patients who developed BM later on was also a relevant finding, calling attention to this protein in primary disease states. The key findings ensuing from this work, schematically depicted in Figure 5, pave the way for future studies directed at the exploitation of MEF2C as a biomarker of BCBM development and prognosis, as well as of the associated signaling pathways as potential therapeutic targets, which will, hopefully, improve the expectancy and QoL of BC patients.

## Figures and Tables

**Table 1:** Patients characterization.

Patients	Primary Tumor	Age	Subtype of Breast Cancer	Number of Brain Metastases	Brain Tumors Size (cm)
1	-	35	TNBC	Multiple	-
2	-	29	TNBC	Solitary	2.8
3	-	78	TNBC	Solitary	2.8
4	-	42	ER+/PR+	Multiple	-
5	-	56	ER+/PR+	Multiple	4–5
6	-	60	ER+/PR+	Solitary	3
7	-	49	ER+/PR+	Multiple	3.8
8	Yes	58	ER+/PR+, HER2+	Multiple	1–2
9	-	67	ER+, HER2+	Solitary	6
10	-	42	ER+/PR+, HER2+	Multiple	-
11	-	41	ER+, HER2+	Multiple	3
12	-	63	ER+/PR+, HER2+	Solitary	4
13	-	48	HER2+	Multiple	-
14	Yes	58	HER2+	Multiple	-
15	Yes	56	HER2+	Multiple	-
16	-	57	HER2+	Multiple	1.5
17	-	46	HER2+	Multiple	5
18	-	41	HER2+	Multiple	-
19	-	51	HER2+	Solitary	3.6
20	-	57	HER2+	Solitary	3.8
21	-	52	HER2+	Multiple	4
22	-	70	-	Solitary	2.6
23	-	59	-	Multiple	-
24	-	70	-	Solitary	3.5

ER+, estrogen receptor positive; PR+, progesterone receptor positive; HER2+, human epidermal growth factor receptor 2 positive; TNBC, triple negative breast cancer.

**Table 2:** Control patients characterization.

Control	Sex	Age	Pathology
1	Male	29	Diffuse Glioma
2	Male	74	Diffuse Glioma
3	Male	35	Diffuse Glioma
4	Female	26	Diffuse Glioma
5	Female	57	Diffuse Glioma
6	Male	31	Diffuse Glioma
7	Male	30	Diffuse Glioma
8	Male	78	Diffuse Glioma
9	Male	27	Diffuse Glioma
10	Female	43	Diffuse Glioma

**Table 3:** Summary of the antibodies and experimental conditions used in immunofluorescence analysis.

Marker	Blocking	Primary Antibody	Dilution	Secondary Antibody	Dilution
Pan Cytokeratin	10% GS + 0.5% Triton X-100	ThermoFisher Scientific, #MA5-12231, Mouse monoclonal	1:500	Alexa Fluor^®^ 488 ThermoFisher Scientific,#A-11001 Goat anti-mouse	1:500
Vimentin	10% GS + 0.5% Triton X-100	ThermoFisher Scientific, #MA3745 Mouse Monoclonal	1:100	Alexa Fluor^®^ 488 ThermoFisher Scientific,#A-11001 Goat anti-mouse	1:500
MEF2C	10% GS + 0.5% Triton X-100	ThermoFisher Scientific, #PA5-28247, Rabbit polyclonal	1:100	Alexa Fluor^®^ 555 ThermoFisher Scientific, #A-21428 Goat anti-rabbit	1:250
Ki-67	3% BSA + 0.5% Triton X-100	ThermoFisher Scientific #PA5-19462, Rabbit	1:100	Alexa Fluor^®^ 555 ThermoFisher Scientific, #A-21428 Goat anti-rabbit	1:500
Ki-67	10% GS + 0.5% Triton X-100	Santa Cruz Biotechnology #sc-7846 Goat Polyclonal	1:100	IgG-FITC Santa Cruz Biotechnology, #sc-2024, donkey anti-Goat	1:500
VEGFR-2	3% BSA + 0.5% Triton X-100	ThermoFisher Scientific, #MA5-15556, Mouse monoclonal	1:250	Alexa Fluor^®^ 488 ThermoFisher Scientific, #A-11001 Goat anti-mouse	1:500
ß-catenin	10% GS + 0.5% Triton X-100	ThermoFisher Scientific, #71-2700, Rabbit polyclonal	1:50	Alexa Fluor^®^ 488 ThermoFisher Scientific, #A-21206 donkey anti-rabbit	1:500

## Data Availability

The data presented in this study are available in the presented figures and supplementary material, as well as in the tables.

## Supplementary Materials

The following are available online at https://www.mdpi.com/2073-4409/10/2/378/s1, Figure S1: Triple negative breast cancer (TNBC) cells express myocyte enhancer factor 2C (MEF2C), which can be silenced using a specific siRNA; Figure S2: Triple negative breast cancer (TNBC) cells express myocyte enhancer factor 2C (MEF2C) and epithelial (pan Cytokeratin) and mesenchymal (vimentin) markers; Figure S3: Triple negative breast cancer (TNBC) cells express Ki-67, vascular endothelial growth factor receptor-2 (VEGFR-2) and β-catenin.
