# Supplementary material for: Myocyte Enhancer Factor 2C as a New Player in Human Breast Cancer Brain Metastases
Source: Cells. 2021 Feb 12;10(2):378. doi: 10.3390/cells10020378 (PMC7917785; doi:10.3390/cells10020378)
Supplement: Supplementary file 1 [file cells-10-00378-s001.pdf]

Myocyte enhancer factor 2C as a new player in 3 human breast cancer brain metastases

Sofia Galego, Linda Azevedo Kauppila, Rui Malhó, José Pimentel and Maria Alexandra Brito

Supplementary Material

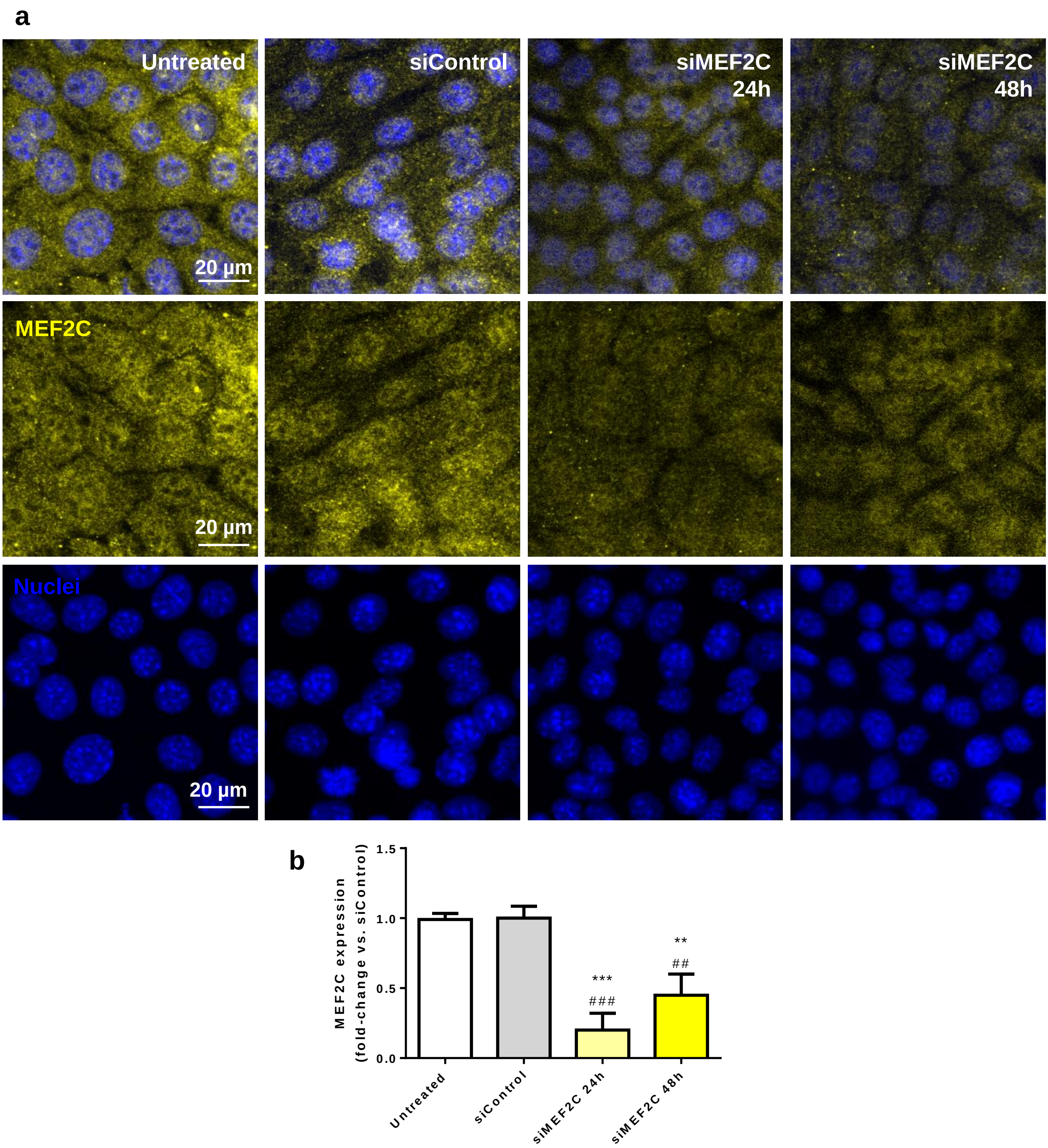

Supplementary Figure 1. Triple negative breast cancer (TNBC) cells express myocyte enhancer factor 2C (MEF2C), which can be silenced using a specific siRNA. Mouse TNBC 4T1 cells were transfected with 10 nM siRNA against MEF2C (siMEF2C), using Lipofectamine™ 3000, for 24 and 48 h and compared with untreated cells (Untreated) and siRNA control (siControl). (**a**) Immunofluorescence analysis of MEF2C (yellow) in 4T1 cells showed its basal expression, particularly in the nuclear region, which is diminished already 24 h post-transfection with siMEF2C. Nuclei were counterstained with Hoescht 33342. (**b**) RT-PCR analysis of MEF2C mRNA in 4T1 cells showed that it is expressed by 4T1 cells, validating the immunofluorescence observations, and highlight its downregulation by siRNA transfection. Statistical differences are denoted with ** $p<0.01$ and *** $p<0.001$ *vs.* Untreated and with $^{\#\#}p<0.01$ and $^{\#\#\#}p<0.001$ vs. siControl. Data are mean ± SEM of three independent biological replicates.

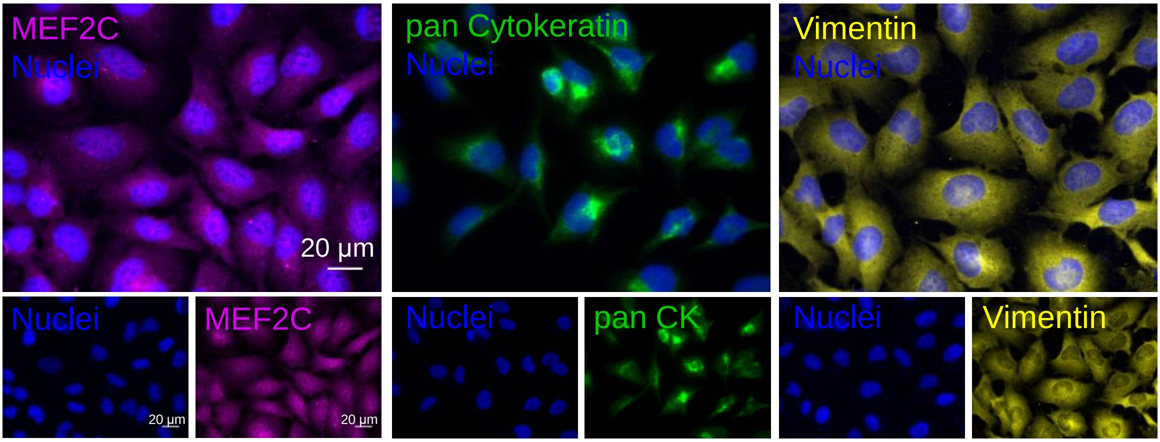

Supplementary Figure 2. Triple negative breast cancer (TNBC) cells express myocyte enhancer factor 2C (MEF2C) and epithelial (pan Cytokeratin) and mesenchymal (vimentin) markers. Cultured human breast cancer cells with brain tropism (MDA-MB-231 Br4) were immunolabelled for each marker and nuclei were counterstained with Hoechst 33342. Representative images are presented.

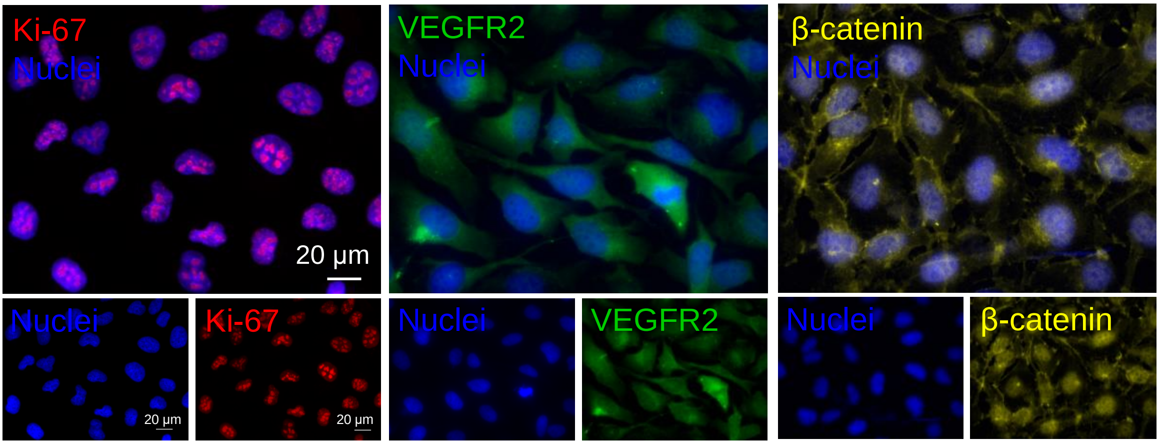

Supplementary Figure 3. Triple negative breast cancer (TNBC) cells express Ki-67, vascular endothelial growth factor receptor-2 (VEGFR-2) and β-catenin. Cultured human breast cancer cells with brain tropism (MDA-MB-231 Br4) were immunolabelled for each marker and nuclei were counterstained with Hoechst 33342. Representative images are presented.
